# What underlies inadequate and unequal fruit and vegetable consumption in India? An exploratory analysis

**DOI:** 10.1016/j.gfs.2019.100332

**Published:** 2020-03

**Authors:** Samira Choudhury, Bhavani Shankar, Lukasz Aleksandrowicz, Mehroosh Tak, Rosemary Green, Francesca Harris, Pauline Scheelbeek, Alan Dangour

**Affiliations:** a Centre for Development, Environment and Policy, School of Oriental & African Studies, London, WC1H 0XG, UK; b Department of Population Health, London School of Hygiene & Tropical Medicine, London, WC1E 7HT, UK

**Keywords:** India, Fruit and vegetable consumption, Nutrition, Food systems

## Abstract

Adequate consumption of fruit and vegetables is key to improved diet-related health in India. We analyse fruit and vegetable consumption in the Indian population using National Sample Survey data. A series of regressions is estimated to characterise the distribution of household fruit and vegetable consumption and explore key socio-economic and food system drivers of consumption. Household income and price are important correlates, but consumption is also higher where households are headed by females, are rural, or involve agricultural livelihoods. Caste is an important source of inequality, particularly amongst those with low consumption, with Scheduled Tribes consuming less F&V than others. We also find preliminary evidence that formal agricultural market infrastructure is positively associated with fruit and vegetable consumption in India.

## Highlights

• Large proportions of the Indian population consume worrying low levels of fruit and vegetables.
• Income and price are key drivers of household consumption.
• Caste is an important source of consumption inequality, particularly amongst those with low consumption.
• Density of wholesale markets (mandis) is positively associated with fruit and vegetable consumption.

## 1 Introduction

Dietary risks are amongst the top risk factors for death and disability in India (Prabhakaran et al., 2018). Fruits and vegetables (F&V) are a key food group providing essential vitamins and minerals, and their intake is particularly important in settings where micronutrient deficiencies are widespread, such as India (Meenakshi, 2016). There are important associations between F&V intake and lowered risk of cancer, cardiovascular disease and all-cause mortality (Aune et al., 2017). This is of particular importance in India, where F&V consumption has a role to play in combating an ongoing crisis relating to diet-related chronic disease (Reddy et al., 2005).

The WHO's Global Strategy on Diet, Physical Activity and Health recommends that per capita F&V consumption (excluding tubers) should exceed 400g/day. However, diets in India are typically cereal-dominated and limited in their diversity (Shankar et al., 2017; Tak et al., 2019). Limited previous research has suggested that consumption of F&V in India has historically been low. Analysis of the 2011-12 National Nutritional Monitoring Bureau data for selected Indian states showed average vegetable consumption amongst men to be 143 g/person/day for men and 138 g/person/day for women (Shankar et al., 2017). A recent analysis of the nationally representative National Sample Survey (2011–2012) indicated that household per capita consumption of F&V is 160 g/person/day for rural India and 184 g/person/day for urban India (Minocha et al., 2018), well short of the WHO benchmark of 400g/person/day.

What might underlie this inadequate level of F&V consumption? On the demand side, low income, high prices and social and geographical inequities are hypothesised as potentially important constraints. Sekhar et al. (2017) found F&V prices to be a major contributor to overall food inflation in India. For example, during 2012–2013, fruit and vegetable price inflation ran at 78% compared to an average for all foods of 18%, with the Indian media highlighting the effects on consumers of an ‘onion crisis’ as onion prices soared by more than 200%. Ruel et al. (2005) noted that F&V consumption is generally expected to be responsive to income growth, but given that F&V are an expensive food source, especially on a per-caloric basis, poorer households struggling to meet energy requirements are likely to find themselves more constrained in increasing consumption (Green et al., 2013; Headey and Alderman, 2019). Regional and social disparities may be important too. Tak et al. (2019) reported that the diversity of diets differs markedly across Indian regions. Previous literature has shown how welfare outcomes in India, including nutrition, can differ substantially across regions and social classifications such as caste, even after controlling for differences in income and other confounders (Cavatorta et al., 2015; Van de Poel and Speybroeck, 2009; Joy et al., 2017).

On the supply side, it has been noted that F&V producers have not responded strongly to increased demand arising from robust economic growth (Pingali, 2015), contributing to high and volatile F&V prices. High transaction costs of linking smallholders to markets and inadequate infrastructure have been identified as major obstacles to producer response (Joshi et al., 2004; Pingali, 2015). Gandhi and Namboodri (2005) characterise F&V value chains in India as highly inefficient marketing structures with poorly coordinated markets and high proportions of spoilage.

However, apart from bivariate associations drawn between F&V intakes and wealth or socio-economic status, studies including F&V as one of many food categories in broader food demand analysis and some insights from small qualitative studies, there is little research examining how F&V consumption in India relates to key economic, socio-demographic or food system drivers. In this paper, we examine the household-level economic, socio-demographic, and key food system drivers of F&V consumption in India. In doing so, we train special focus on the lower tail of the F&V distribution with the worst consumption outcomes.

## 2 Data and methods

### 2.1 Data

Our primary source of data is the 68th (latest available) round of the nationally representative cross-sectional survey on household expenditure and consumption, the National Sample Survey (NSS), conducted in 2011–2012. The NSS Household Consumption Expenditure Survey records both quantity (purchase + home production) and value of food items at household level. Information on the NSS's stratified multistage sampling design has been reported elsewhere (Government of India, 2010. Unlike the National Family Health Survey (NFHS) datasets which do not provide information on quantity of food items purchased, the NSS 2011–12 offers two alternatives for the computation of food consumption measures. One alternative, called ‘type 1 data’, has a recall period of 30 days and has been used in previous computation of summary statistics of F&V consumption (Minocha et al., 2018). Our analysis is based on an alternative NSS survey format (‘type 2’), which uses a reference period of 7 days preceding the survey and retains the 30 day recall only for some food items (cereals, pulses and sugar). We use the type 2 schedule based upon 7 day recall since using a shorter recall period can potentially help improve accuracy, particularly for nutrient-rich food groups, compared to the 30 day recall period (Aleksandrowicz et al., 2017). The NSS 2014 report on the 68th round also examines some differences between Type 1 and Type 2, and these are summarised by Aleksandrowicz et al. (2017) that there was a small increase in overall calories in Type 2 versus Type 1, and higher intake of those items that used the 7 day recall (meats, eggs, fruit, veg, etc). The original NSS sample consists of 101,651 (59,683 rural and 41,968 urban) households. Following exclusion of households with extreme values of^1^ per capita calorie intake, our final primary estimation sample consists of 98,868 households.

Our dependent variable is household per-capita fruit and vegetable consumption (g/capita/day), which is based on the sum of fruits and vegetables (excluding potato)^2^ consumed in the home by the household in the previous 7 days from a single respondent, usually the female adult of the household who recalls other household members’ consumption. Quantity of consumption from purchased and own production are asked separately and we have taken the sum of these as the household intake. The survey asks about quantities produced/purchased for about 140 individual food items, with a small number of questions on meals/snacks eaten out of home. We aggregated the individual fruit and vegetable items into our fruit and vegetable categories. The fruit and vegetable groups include mango, orange, guava, banana, papaya, grapes, melon, other fruits, onion, garlic, leafy vegetables, tomato, gourd, carrot and other vegetables. When calculating consumption, adjustments have been made to include (1) meals prepared at home but consumed by non-members and (2) meals received for free from other households by household members. Household per capita consumption is calculated by dividing the total household F&V consumption by household size (household composition is controlled for as a covariate in the regression analysis). We use a simple division by household size in order to maintain consistency with the key previous literature (Minocha et al., 2018), and also in accordance with practice in the economics literature that uses NSSO food consumption data (eg. Deaton and Dreze, 2009). However, as an alternative we also provide a full set of results in the online appendix that normalises on the basis of adult equivalent units. Although consumption is normalised by the number of household members here, it must be kept in mind that this remains a household–level measure and is only meant to be a proxy for, rather than an attempt at individual consumption measurement.

Our set of explanatory variables comprises a variety of household level economic and socio-demographic indicators and food systems level factors that have been associated with household dietary outcomes in previous literature (Stifel and Minten, 2017; Alderman and Headey, 2017; Ruel et al., 2005). To proxy income or purchasing power, we include per capita monthly expenditure. We proxy prices using unit values, *i.e.* by using the ratio of expenditure over quantity (in doing so, it is recognised that unit values incorporate a quality choice dimension). Thus, for a composite good such as fruit and vegetables, unit values, *i.e.* expenditure divided by quantity consumed, will reflect household choices both about individual types of F&V consumed, and also about relative consumption of higher or lower grade of produce. Therefore, caution is warranted in the interpretation of regression coefficients. Two such unit value measures are calculated for each household, one for all foods and one for the category of fruit and vegetables. The unit value of fruit and vegetables divided by the unit value of all foods is then used as the proxy relative price of fruit and vegetables in all regressions. Caste is represented by a set of dummy variables, where Scheduled Tribes, Scheduled Castes and Other Backward Castes are measured against the baseline of ‘Other/Upper’ castes.^3^ We include a set of state level dummy variables in all regressions to control for regional heterogeneity. In addition to the above, a measure of education is included to proxy nutritional knowledge, specified as years of schooling of the household head (Webb and Block, 2004). Research on intrahousehold allocation of resources suggests that men and women do not necessarily pool their resources and hence may allocate resources differently, depending on their bargaining power within a household (Alderman et al., 1995; Hoddinott and Haddad, 1995). Therefore, we include a dummy variable indicating female headed households in our analyses.

In order to capture socio-cultural aspects beyond what is controlled for by state-level fixed effects, we also include a binary variable to indicate whether a household is Hindu or not.^4^ There is now considerable evidence that in the presence of market failures in developing countries, households are highly dependent on own production (Sibhatu and Qaim, 2018), and accordingly we include variables to represent whether a household is rural or urban, and whether its primary employment is in agriculture. Since adult and child consumption levels are likely to differ with implications for household per capita computations, we include the number of children in the household as a covariate.

In addition to our main analysis, we also conduct supplementary exploratory work to gauge the associations of two key supply-side variables in the form of road infrastructure and the density of state-run agricultural markets with F&V consumption. Roads and markets are potentially critical constraints to the distribution of fruit and vegetables and therefore to their availability and prices across the country, particularly since cold chain availability is minimal to non-existent in many parts of India, and produce is largely traded as fresh (Desai, 2011). A high proportion of fruits and vegetables in India is transported via trucks, often across hundreds of kilometres, to be sold to traders at large state-run *mandis* (wholesale markets). The NSSO dataset itself does not contain information on such infrastructure variables. However, for a proportion (approximately 20%) of the overall NSSO sample, we are able to match the district location of the household with district-level information on roads and markets. These district-level data on roads and agricultural markets have been retrieved from Village Dynamics in South Asia (VDSA) project of the International Crops Research Institute for the Semi-Arid Tropics (ICRISAT). Such information from the VDSA is available for a subset of 53 districts in 2010–11. No claims can be made about potential randomness in whether district level data are missing or not and therefore this additional analysis should be considered an initial and partial exploration. Road density is defined as road length per 1000 km^2^ of geographical area. Market density is calculated as the number of formal agricultural markets per 1000 km ^2^of geographical area.

### 2.2 Methods

We start with a set of data visualisations, first graphing the distribution of F&V consumption in the population, and then applying non-parametric regressions in the form of local polynomial smoothers to assess non-linear bivariate relationships between household F&V consumption and a core set of covariates. We then use ordinary least squares regression models to assess the associations between fruit and vegetable consumption and household-level economic and socio-demographic variables using the full sample.

Subsequently, we turn attention to the question of inequality in F&V consumption, specifically asking how the influence of key covariates on F&V consumption varies across the F&V consumption distribution. One option available for such an approach would be a categorical dependent variable model such as a probit or logistic regression, say based on grouping F&V consumption into ‘low’, ‘medium’ and ‘high’. However, in addition to the *ad-hoc* nature of such grouping, this would also entail a loss of statistical information and a restrictive characterisation of the joint distribution of the outcome and the covariates (Zanello et al., 2016). We apply Unconditional Quantile Regressions (UQR), specifically Firpo et al.'s (2010) unconditional Recentred Influence Function (RIF) UQR method. The RIF regression methods allow us to estimate the unconditional quantile effects of the covariates on F&V consumption at any quantile of the distribution. Unlike routinely applied conditional quantile regression methods where the estimated relationship between covariate and outcome is conditional on the values of other covariates, Firpo et al.'s method provides unconditional estimates, and has been applied in the analysis of food and nutrition outcomes by Zanello et al. (2016) and Jolliffe (2011) among others. Here, we estimate and present UQR results for the 10th, 25th, 50th, 75th and 90th percentiles of F&V consumption.

Finally, we carry out our exploratory analysis on the smaller sample with available information on road and market density, to gauge the relationship between infrastructure and F&V consumption. Since the regional-level infrastructure variables would conceptually influence household F&V consumption primarily via their prices, our set of covariates for this exercise include all the household-level variables described above, except for price, along with market and road density. The infrastructure variables are at the district level, whereas the rest of the covariates are at household level. Frequently applied approaches in such settings include ignoring the hierarchical structure of the data and assigning district-level values to households, or carrying out analysis based on district level averages. Instead, we maintain and recognise the hierarchy in the data and estimate a multi-level model comprised of two levels, household and district. Specifically, we estimate a random intercept model (Raudenbush and Byrk, 2002) that allows the identification of the influence of the district-level infrastructure variables on household F&V consumption whilst allowing the random error term to vary by district.

## 3 Results

### 3.1 *Descriptive results* and bivariate relationships

Table 1 reports summary statistics for all the variables used in the analysis. The WHO norm of 400 g/person/day for F&V refers to adult individuals whereas the consumption outcome here is derived from household level data. Therefore, we refer instead to the benchmark household-level adequacy indicator of 400 g/person/day referred to in the FAO-World Bank ADePT-FSM (Moltedo et al., 2014) and discussed in INDDEX Project (2018). The median household per capita consumption of 200 g/person/day (Table 1) is far short of the 400 g/person/day benchmark for household level per capita consumption of fruit and vegetables. It is worth noting that this computation based on 7-day recall is larger than the 160 g/person/day for rural India and 186 g/person/day for urban India reported by Minocha et al. (2018) from the same survey using 30-day recall.

**Table 1:** Summary statistics.

Variable	Mean	Median	SD
*Household level indicators (NSSO data)*			
Household F&V consumption (g/capita/day)	229.9	199.82	141.51
Household Vegetable consumption	159.16	139.93	95.09
(g/capita/day)	70.74	47.14	92.3
Household Fruit consumption (g/capita/day)			

Per capita monthly expenditure (Rs)	1950.37	1462.13	1897.4
Relative price of F&V^2^	1.94	1.76	1.29
Household size	4.52	4.00	2.14
Number of children under 5 (%)	46.74	0.00	78.89
Household head years of education	5.54	6.00	3.67
Female headed households (%)	11.19	0.00	31.53
Rural location (%)	69.73	1.00	45.94
Agricultural households (%)	50.66	1.00	50
Hindu (%)	83.03	1.00	37.54
Scheduled Tribes (%)	8.90	0.00	28.47
Scheduled Castes (%)	19.15	0.00	39.35
Other Backward Classes (%)	43.19	0.00	49.53
Other/UpperCastes (%)	28.76	0.00	45.27
*District level indicators (VDSA data)*			
Road density (km of road per 1000 km sq. land area)	0.69	0.69	0.34
Market density (number of agricultural	3.06	2.59	2.17
markets per 1000 km sq. of land area)			

**Fig. 1:**
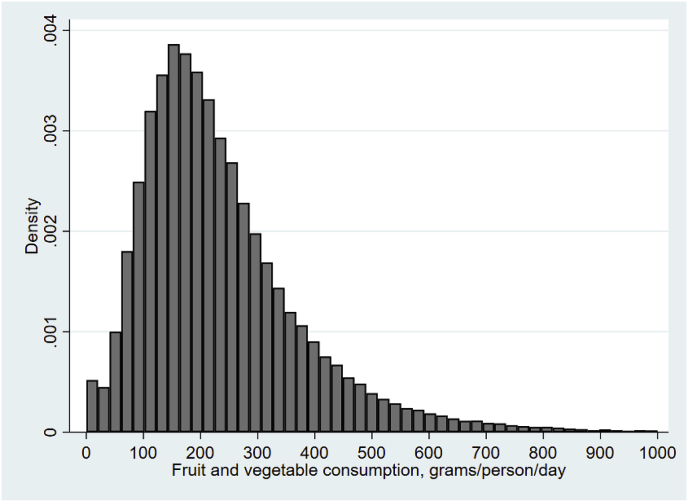
Distribution of Fruit and Vegetable (F&V) consumption (g/capita/day) Source: NSS (2011– 2012).

Fig. 1 shows that the distribution of consumption in the sample is highly unequal. There are considerable proportions of households with consumption less than 100 g/person/day and even 50 g/person/day. There is also a long tail of households consuming more than 400 g/person/day. Online appendix A2 provides summary statistics for this group. It is evident from comparing that table with Table 1 for the overall population above that households consuming in excess of the benchmark are substantially richer, and also on average have smaller household sizes and more educated household heads.

We turn to bivariate relationships between F&V consumption and key covariates of interest: caste, income and prices. Table 2 highlights another aspect of inequality in F&V consumption in India, connected to caste. Policy structures in India have long recognised four broad caste groupings reflecting the extent of socio-economic disadvantage in descending order: Scheduled Tribes, Scheduled Castes, Other Backward Castes and ‘Other/Upper’ Castes. Table 2 shows that F&V consumption tracks this caste grouping, with average group consumption lowest among Scheduled Tribes and rising to highest among ‘Other/Upper’ Castes. ‘Other/Upper’ castes on average consume 60 more g/person/day, (or 34%) F&V than Scheduled Castes.

**Table 2:** Fruit and vegetable consumption by caste category.

Social group	Number of observations	Mean F&V consumption (g/person/day)	Standard Deviation
Scheduled Tribes	13,356	187.90	120.90
Scheduled Castes	15,594	204.30	126.55
Other Backward Classes	39,268	227.10	136.13
Others	32,028	251.90	163.03

**Fig. 2:**
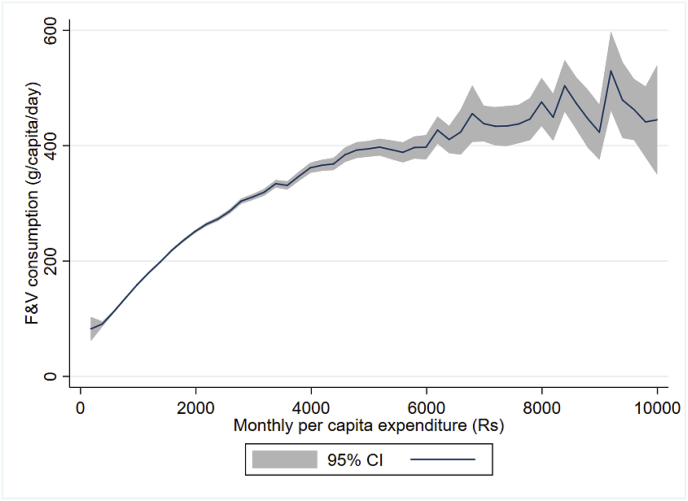
Nonparametric estimates of the relationship between F&V consumption and monthly per capita expenditure (Rs) Local polynomial smoothing estimates with 95% confidence intervals: regression fitted line in bold; confidence interval in grey shade. Source: NSS (2011– 2012). (For interpretation of the references to colour in this figure legend, the reader is referred to the Web version of this article.)

**Fig. 3:**
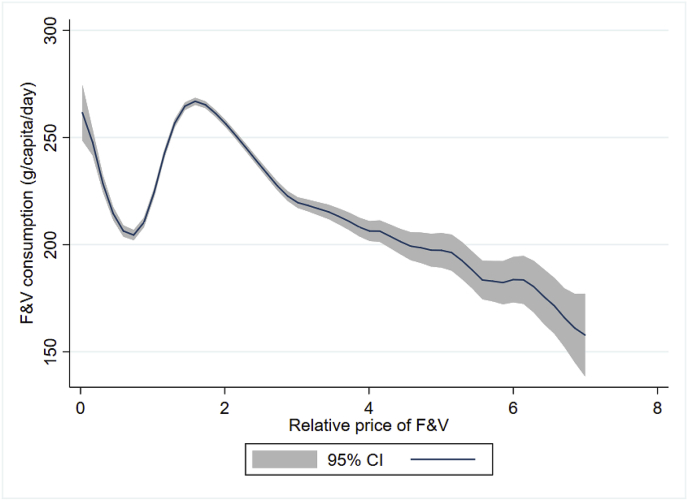
Nonparametric estimates of the relationship between F&V consumption and relative price of F&V Local polynomial smoothing estimates with 95% confidence intervals: regression fitted line in bold; confidence interval in grey shade. Source: NSS (2011– 2012).. (For interpretation of the references to colour in this figure legend, the reader is referred to the Web version of this article.)

Fig. 2, Fig. 3 graph bivariate relationships between F&V consumption and the two key variables, income/expenditure and prices. Fig. 2 demonstrates the clear positive gradient between F&V consumption and household per-capita expenditure as a proxy for income. Evidently, the bivariate relationship demonstrates some non-linearity and the income effect appears to level off at high incomes. In Fig. 3, a steep decline of F&V consumption with relative F&V price is observed for the most part. However, the bivariate relationships are only an initial guide, and are potentially confounded by numerous other changes. We now focus attention on regression results that control for such confounding.

### 3.2 OLS regression results

We first report results based on ordinary least squares (OLS) estimates (Table 3) using the full sample.^5^ The model includes a full set of state dummy variables to provide control for unobserved regional and state policy influences that may impinge upon household F&V consumption (Cavatorta et al., 2015). The results in Table 3 confirm a strong association between household per capita expenditure and F&V consumption. Based upon the estimated coefficient, an income elasticity of 0.65 can be calculated at the mean F&V consumption in the sample (meaning a 10% increase in income is associated with a 6.5% increase in household per-capita F&V consumption). This estimate is consistent with an F&V income elasticity range between 0.60 and 0.97 calculated by Ruel et al. (2005) for a range of African countries. The coefficient on the price (proxied by unit value) of F&V relative to all foods is also significant at the 1% level, and negative as expected, indicating that higher F&V prices do play a role in discouraging consumption.

**Table 3:** OLS regression for household fruit and vegetable consumption (g/capita/day).

	F&V consumption (g/capita/day)
Log per capita monthly consumer expenditure	129.72***
	(2.28)
Log relative price of F&V	−14.55***
	(2.52)
Household size	−11.19***
	(0.38)
Number of children under 5	0.80
	(0.71)
Household head years of education	−0.48**
	(0.24)
Female headed households	15.88***
	(2.67)
Rural location	15.53***
	(1.97)
Agricultural households	6.21***
	(1.33)
Hindu	2.11
	(2.13)

**Caste (baseline: ‘other’ caste)**	
*Scheduled Tribes*	0.84
	(2.77)
*Scheduled Castes*	−1.90
	(2.22)
*Other Backward Classes*	−0.01
	(1.95)
Observations	98,868
R-squared	0.36

Standard errors in parentheses ***p < 0.01, **p < 0.05, *p < 0.1. Covariate set includes state dummy variables.

Also noteworthy in Table 3 are the positive and statistically highly significant coefficients relating to rural status and agricultural occupation of the household. A rural location is associated with a 15 g/person/day higher F&V consumption, all else held equal, while being occupied in the agriculture sector is associated with a 6 g/person/day increase. Thus, an urban disadvantage in F&V consumption is observed once higher incomes typically observed in urban areas are controlled for. This suggests that market failures may be at play. Where markets are complete and efficient, engaging in or being proximate to agricultural production should have no relationship with F&V consumption, once income and prices are controlled for. However, market failures may lead to a direct link between agricultural involvement, or being in proximity to agricultural production, and improved F&V consumption.^6^ This is consistent with a recent literature emphasising agricultural production and nutrition linkages among farm households in South Asia arising from market failures (Shankar et al., 2019).

F&V consumption in female headed households is higher by about 15 g/person/day compared to male-headed households after controlling for other covariates. Diseconomies of scale are observed in household F&V consumption in Table 3, with larger households linked with lower F&V household consumption per capita. The small and statistically insignificant coefficients attached to the caste variables (in comparison to the baseline of ‘Other/Upper’ caste) are very informative. Considered in conjunction with the sizeable differences observed in F&V consumption across caste groups in Table 2, they suggest that caste-based inequality in F&V consumption arises from differential levels of income and other observed covariates of consumption across castes.

### 3.3 Unconditional quantile regression results

Table 4 presents results from unconditional quantile regressions (UQR) for the 10th, 25th, 50th, 75th and 90th quantiles. The UQR share an identical set of covariates (including state dummy variables) with the OLS regressions discussed in Table 3, and offer insight into how relationships differ across the F&V consumption distribution rather than just at the mean (OLS). Although the coefficients attached to the income and relative price variables increase along the F&V consumption distribution, note that the semi-log functional form means that the coefficients by themselves do not directly indicate strength of association. The implied income elasticity declines from 1.2 at the 10th percentile to 0.6 at the 90th percentile of consumption. Thus, F&V consumption does indeed respond substantially to income improvements amongst those consuming the least.

**Table 4:** RIF Unconditional Quantile Regression Results of drivers of F&V consumption (g/capita/day).

	(1)	(2)	(3)	(4)	(5)
Quantiles of the F&V consumption distribution					
	10th	25th	50th	75th	90th
Log per capita monthly consumer expenditure	52.273***	71.282***	105.593***	160.040***	238.539***
	(1.620)	(1.382)	(1.610)	(2.596)	(5.365)
Log relative price of F&V	0.738	4.405*	−2.611	−17.209***	−48.038***
	(2.694)	(2.286)	(2.510)	(3.583)	(5.983)
Household size	−2.343***	−5.153***	−9.720***	−15.907***	−20.764***
	(0.382)	(0.356)	(0.401)	(0.598)	(1.130)
Number of children under 5	−1.602	−2.791***	−1.898*	0.372	6.227***
	(1.120)	(1.004)	(1.019)	(1.260)	(1.802)
Household years of education	−0.099	0.159	−0.502**	−0.461	−1.380**
	(0.215)	(0.204)	(0.240)	(0.363)	(0.681)
Female headed households	−0.525	1.661	6.194**	18.735***	42.736***
	(2.267)	(2.021)	(2.460)	(3.841)	(7.280)
Rural location	5.609***	9.518***	11.438***	18.801***	27.408***
	(1.202)	(1.220)	(1.588)	(2.562)	(4.859)
Agricultural households	3.877***	6.113***	4.626***	5.727***	5.770
	(1.322)	(1.220)	(1.449)	(2.191)	(3.965)
Hindu	−0.144	0.098	2.768	7.203**	7.925
	(1.777)	(1.688)	(1.985)	(2.957)	(5.213)
Scheduled Tribes	−10.107***	−8.505***	−0.640	4.613	10.442
	(3.096)	(2.794)	(3.071)	(4.186)	(6.878)
Scheduled Castes	−1.265	−1.682	−4.729**	−4.329	−2.981
	(2.133)	(1.975)	(2.315)	(3.441)	(5.997)
Other Backward Classes	2.995*	2.681*	0.890	−0.994	−0.311
	(1.643)	(1.556)	(1.932)	(2.990)	(5.537)

Observations	98,868	98,868	98,868	98,868	98,868
R-squared	0.115	0.208	0.270	0.257	0.175

However, a striking pattern apparent from Table 4 is that several of the key covariates of F&V consumption, such as F&V relative prices and the gender of the household head, actually have weaker relationships with F&V consumption at the lower tail than they do higher up in the distribution. The relative price coefficient is only statistically significant at the higher quantiles of the F&V consumption distribution. An association between household head gender and F&V consumption is practically absent among low consumption households, whereas gender associations strengthen along the top half of the distribution to make a 42 g/person/day difference at the 90th percentile. Likewise, the positive association of rural location with F&V consumption strengthens five-fold when moving from the 10th to the 90th percentile of consumption. Household size makes only a small difference at low consumption levels, but has more sizeable implications at the top of the distribution. Taken together, this pattern of weak relationships in the lower quantiles suggests that F&V consumption of the lower tail may be challenging to shift via identification of typical policy levers or specific groups to focus interventions on.

There is one association relating to caste in Table 4 that is stronger at the lower tail than in the rest of the distribution. The OLS results indicated that, once income and other covariates are controlled for, caste makes little difference to F&V consumption. The UQR results suggest to the contrary that there *is* a negative Scheduled Tribe association with F&V consumption at the lower tail, even after control for income and other confounders. This effect disappears in the upper half of the distribution, resulting in the overall insignificant OLS estimate observed earlier. All else held equal, a household at the 10th percentile of F&V consumption and classified as belonging to a Scheduled Tribe has a 10g/person/day lower F&V consumption compared to a household from Other/Upper castes in that percentile. Specifically, this points to the need to address a caste-based inequality that exacerbates very low consumption levels. With respect to methods, this underscores the importance of allowing regression coefficients to vary across the outcome distribution in such settings.

### 3.4 Multilevel regression results

Finally, in Table 5, we provide a summary of the results of our exploratory analysis based on the smaller sample for which district-level road and market infrastructure information is available. In the first column, we present coefficients relating to the infrastructure variables from a model based on OLS with state-level dummy variables, and in the second column we show estimates for infrastructure from the multi-level random intercept model. Note that the OLS model controls for cross-sectional heterogeneity via *state* level fixed effects, given district-level effects are not separately identified from the infrastructure variables measured at the district level. The multi-level model, on the other hand, controls for cross-sectional heterogeneity via *district* level random effects. The estimates do not reveal consistent evidence for the influence of road infrastructure on F&V consumption – the OLS fixed effects model shows a statistically significant positive coefficient while the multilevel model produces a statistically insignificant coefficient. However, both models suggest a small albeit positive and statistically significant relationship between district-level density of formal agricultural markets and F&V consumption. Although data deficiencies imply that this result should be interpreted with caution, these preliminary estimates suggest that further analysis based on more complete VDSA data when available may be worthwhile.

**Table 5:** Multilevel regression estimates of influence of district-level road and market infrastructure on household F&V consumption (grams/person/day).

	(1)	(2)
	OLS with state dummy variables	Multilevel regression
Road density (km of road per 1000 km sq. of land area) ***	12.00***	−13.90*
	(4.09)	(5.59)
Market density (number of agricultural markets per 1000 km sq. of land area)	2.24***	4.09***
	(0.59)	(0.76)

Observations	13,402	13,402

## 4 Discussion and conclusion

Dietary risks constitute the second most important risk factor for death and disability in India, following malnutrition, and in the decade from 2007 to 2017 the contribution of dietary risks to disability adjusted life years in India increased by 35% (Prabhakaran et al., 2018). A recent major prospective cohort study, PURE (Prospective Urban Rural Epidemiology), presented rare evidence from LMIC settings, including India, for the health implications of F&V intake (Miller et al., 2017). Discussing this evidence, the authors argued that “modest” levels of consumption are sufficient to provide high benefits, noting, “… *even three servings per day (375 g/day) show similar benefit against non-cardiovascular and total mortality as higher intakes* …” (Miller et al., 2017, p. 2047). Yet, in India, as also highlighted by Minocha et al. (2018), average consumption is well short of these modest targets. In this paper, we have sought to conduct an initial examination of the socio-demographic and economic basis of household F&V consumption in India.

*Summary of results:* To summarise the key results: firstly, not only is the average consumption low, but consumption is also highly unequal, with large proportions of households displaying worryingly poor consumption levels. Secondly, as expected, household income and relative F&V price emerge as important correlates of F&V consumption, but household F&V consumption is also higher where households are headed by females, are rural, or involve agricultural livelihoods, all else being equal. Thirdly, the association of F&V consumption with covariates is seen to vary substantially across the consumption distribution. While the consumption of households with the poorest consumption levels responds most strongly to income growth, many of the other important covariates such as gender, rural location and relative prices display strongest associations at the *top* of the F&V consumption distribution. Fourthly, caste emerges as an important locus of inequality, mirroring patterns reported with respect to other population welfare outcomes in India. The distribution of F&V consumption across caste categories shows that Scheduled Tribes, long-recognised as the most socio-economically disadvantaged group in India, consume less F&V than others, particularly the ‘Other/Upper’ caste group. The mean (OLS) regression suggests that this is largely a matter of an income disadvantage. Yet, the quantile regression analysis demonstrates that amongst those with the worst consumption levels, there is indeed a further disadvantage faced by Scheduled Tribes even after controlling for income and other confounders.

We have also found some preliminary evidence that formal agricultural market infrastructure is positively correlated with F&V consumption in India. Note that the VDSA data used here on market density is restricted to formal state-regulated wholesale markets (*mandis*). These public wholesale markets were set up in the 1960s with the expectation that all agricultural trade must flow through them, thereby restricting the exploitative nature of wholly private food trade. Although subsequent reforms to the Agricultural Produce Market Committee (APMC) have provided impetus for private trade, the regulated public markets remain the mainstays of agricultural marketing in India, however, and our results (including control for state-level heterogeneity) suggest they have a role to play in bolstering F&V consumption. Chatterjee et al. (2017) find for India that an increase in such formal markets induces competition for farmer's produce and thereby lead to better returns to farmers (as compared to intermediaries). An implication is that higher market density has a role to play in improving farmer supply of F&V. Plausibly, a greater density of formal markets also has a role to play in more equitably distributing produce across consuming regions.

*Policy implications:* Private-sector led downstream change in F&V value chains in India is occurring in the form of the expansion of supermarkets and modern retail (Reardon and Minten, 2011). However, the relevance of such transformation to F&V consumption of the poor is questionable, and government policy remains the key lever for broad-based change. Policies to improve F&V consumption in India are almost exclusively focused on production and upstream parts of F&V value chains, even though improving supply is only one element in improving F&V consumption.

Following a review of the policy environment for F&V in India, Khandelwal et al. (2019) concluded that not only did agricultural policy relating to F&V focus almost exclusively on economic opportunities for producers, largely ignoring consumer nutritional considerations, but that even the National Nutrition Policy contained few concrete proposals to improve F&V intakes. The National Food Security Act makes provision for cereals for disadvantaged consumers, but not for F&V (Government of India, 2013; Thow et al., 2018).

Our research suggests on a positive note that continuing household income growth in India will improve F&V consumption, particularly amongst those consuming the least. However, given the large consumption deficit compared to the norm and the inequality inherent in relying on income growth, there is also an urgent need for downstream policies closer to the consumer. For example, government-provided nutrition schemes for nutritionally vulnerable sections, such as the Mid-day Meal Scheme in government schools, have the potential to incorporate more F&V provision. Nakao and Tsuno (2018) show that the Mid-Day Meal Scheme's focus on food grain provision means that F&V provision is minimal. Mainstreaming nutrition education in school curricula and at Integrated Child Development Services centres has also been identified as an important avenue for improving F&V consumption in the long term (Thow et al., 2018). Since income is one of the few variables to exert an influence on the lower tail of F&V consumption, income transfers may offer potential as a policy intervention option. Our results also suggest that all policies will need to tailor strategies to reach Scheduled Tribes in particular, in line with previous literature documenting the numerous barriers faced by this section in accessing public services relevant to nutrition (Thorat and Sadana, 2009). Basic income continues to be a hotly debated topic in India. However, pilot interventions such as the SEWA-Unicef cash transfer scheme that included tribal villages have shown promise with regard to nutrition-related outcomes (Desai and Vanneman, 2016), and may hold potential for improving F&V intake as well.

*Limitations and future research:* It is worth emphasising that this analysis is of a preliminary and exploratory nature, aiming to focus attention on the important topic of F&V consumption in India, rather than an attempt to establish definitive estimates or causal relationships. A number of drawbacks are recognised, including the cross-sectional nature of the data, the single equation (rather than demand system) approach to estimation, measurement issues including using unit values as proxy for prices, and the household-level nature of the data and analysis that stops short of the individual level perspective typical in the health literature.

Given the number of people with inadequate F&V consumption in India and the importance of F&V consumption to multiple major health outcomes in the country, a broad research agenda focused on improving the availability, affordability and consumption of F&V across the entire population is called for. Following on from the research reported here, the role of market and road infrastructure in improving F&V availability, particularly for poorer sections of the population, is an important area for further investigation. Our research has also suggested special focus on the constraints faced by Scheduled Tribes in accessing F&V. There is also a need to understand how F&V consumption has changed over time and the drivers of such change, in order to obtain a dynamic perspective. Urgent research questions also arise about how various F&V policies and interventions in India, ranging from F&V aggregation and marketing schemes for smallholder producers, to value chain interventions, can be made more nutrition-sensitive and focused on the needs of poorer consumers.

## Funding

This study forms part of the Sustainable and Healthy Food Systems (SHEFS) programme supported by the Wellcome Trust's Our Planet, Our Health programme [grant number: 205200/Z/16/Z]. Funding body had no role in the data collection, analysis or interpretation, and no role in the study design or in writing the manuscript.

## Footnotes

1 Based on overall dietary energy intake, we calculated outliers corresponding to values beyond 2.5 SDs around the mean. Correspondingly, we dropped households with per capita calorie intakes less than 50 and greater than 4300.

2 Tubers are excluded in the WHO recommendation of 400 g of F&V/day.

3 The official terminology is “Other”.

4 Appendix Table A1 also shows results of Table .3 excluding religion.

5 Variance Inflation Factors (VIF) were computed to check for multicollinearity. All VIFs were less than 2, considerably smaller than benchmark values, and thus there is no evidence of substantial collinearity in the data.

6 Of course, urban markets for F&V are also often weak in India, particularly in low income areas and especially given lack of cold chain availability. However, F&V availability arising from own production or proximity to production is likely to be more limited in urban than in rural areas.
