# Supplementary material for: What underlies inadequate and unequal fruit and vegetable consumption in India? An exploratory analysis
Source: Glob Food Sec. 2020 Mar;24:100332. doi: 10.1016/j.gfs.2019.100332 (PMC7063694; doi:10.1016/j.gfs.2019.100332)
Supplement: Multimedia component 1 [file mmc1.docx]

Appendix Table S1. Summary statistics

| Variable | Mean | Median | SD |
| --- | --- | --- | --- |
| *Household level indicators (NSSO data)* |  |  |  |
| Household F&V consumption (g/adult equivalent/day) | 288.69 | 247.01 | 188.52 |
| Household Vegetable consumption | 199.64 | 172.66 | 126.3 |
| (g/adult equivalent/day) |  |  |  |
| Household Fruit consumption (g/adult equivalent/day) | 89.04 | 57.11 | 119.5 |
|  |  |  |  |
| Per capita monthly expenditure (Rs) | 1950.37 | 1462.13 | 1897.4 |
| Relative price of F&V^2^ | 1.94 | 1.76 | 1.29 |
| Household size | 4.52 | 4.00 | 2.14 |
| Number of children under 5 (%) | 46.74 | 0.00 | 78.89 |
| Household head years of education | 5.54 | 6.00 | 3.67 |
| Female headed households (%) | 11.19 | 0.00 | 31.53 |
| Rural location (%) | 69.73 | 1.00 | 45.94 |
| Agricultural households (%) | 50.66 | 1.00 | 50 |
| Hindu (%) | 83.03 | 1.00 | 37.54 |
| Scheduled Tribes (%) | 8.90 | 0.00 | 28.47 |
| Scheduled Castes (%) | 19.15 | 0.00 | 39.35 |
| Other Backward Classes (%) | 43.19 | 0.00 | 49.53 |
| Forward Castes (%) | 28.76 | 0.00 | 45.27 |
| *District level indicators (VDSA data)* |  |  |  |
| Road density (km of road per 1000 km sq. land area) | 0.69 | 0.69 | 0.34 |
| Market density (number of agricultural | 3.06 | 2.59 | 2.17 |
| markets per 1000 km sq. of land area) |  |  |  |

Source: NSS (2011-2012), VDSA (2011-2012). Note: the sample size for the NSSO data is 98,868 households. The VDSA data are for 53 districts that cover 23,847 of the households in the NSSO sample. ^2^ Relative price of F&V: price (unit value) of fruits and vegetables relative to all food.

Appendix Fig S1. Distribution of Fruit and Vegetable (F&V) consumption (g/adult equivalent/day)

Source: NSS (2011-2012)


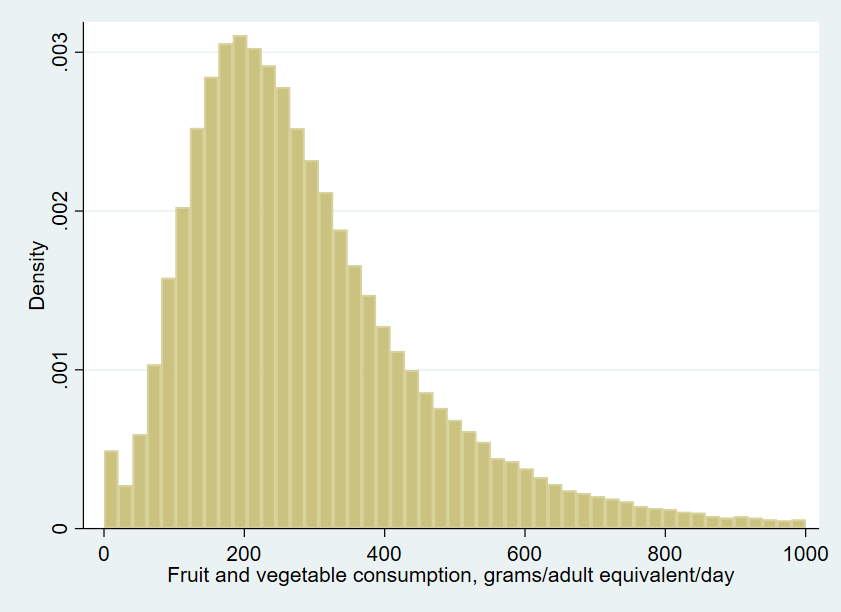


Appendix Table S2. Fruit and Vegetable Consumption by Caste Category

| **Social group** | **Number of observations** | **Mean F&V consumption (g/adult equivalent/day)** | | **Standard Deviation** |
| --- | --- | --- | --- | --- |
| Scheduled Tribes | 13,356 | 233.61 | | 155.30 |
| Scheduled Castes | 15,594 | 255.90 | | 167.54 |
| Other Backward Classes | 39,268 | 285.44 | | 181.99 |
| Others | 32,028 | 316.13 | 217.19 | |

Source: NSS (2011-2012)

Appendix Fig S2. Nonparametric estimates of the relationship between F&V consumption and monthly per capita expenditure (Rs)


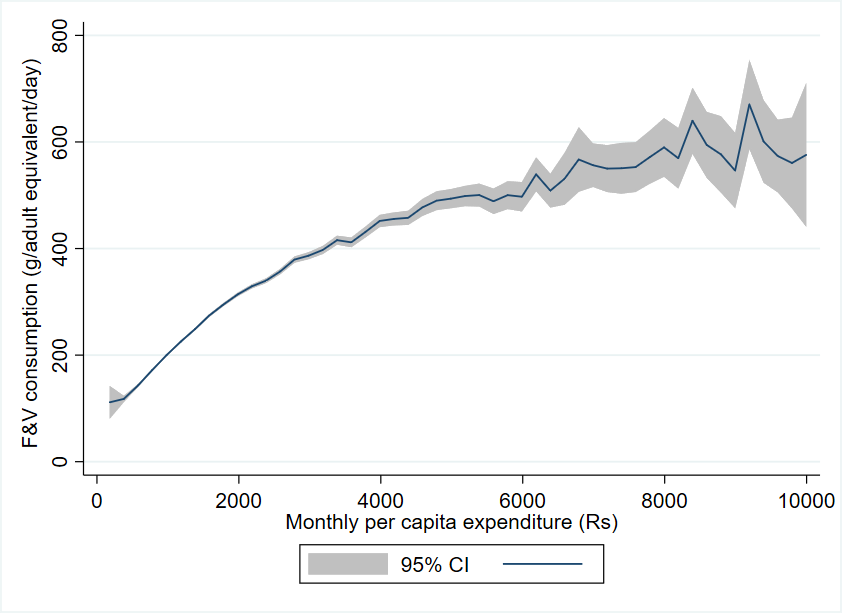


Local polynomial smoothing estimates with 95% confidence intervals: regression fitted line in bold; confidence interval in grey shade. Source: NSS (2011-2012)

Appendix Fig S3. Nonparametric estimates of the relationship between F&V consumption and relative price of F&V


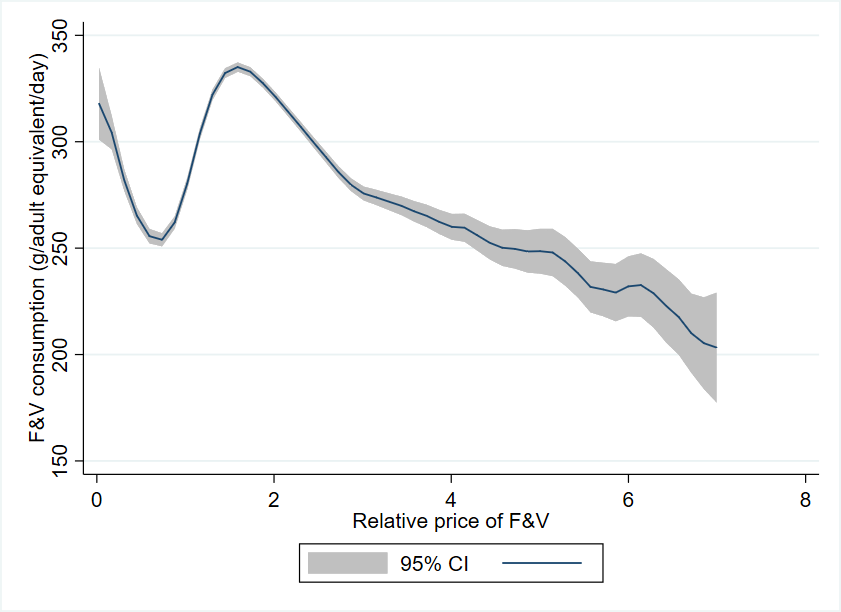


Local polynomial smoothing estimates with 95% confidence intervals: regression fitted line in bold; confidence interval in grey shade. Source: NSS (2011-2012)

Appendix Table S3. OLS Regression for Household Fruit and Vegetable Consumption (g/adult equivalent/day)

|  |  |
| --- | --- |
|  |  |
|  | **F&V consumption (g/adult equivalent/day)** |
|  |  |
| Log per capita monthly consumer expenditure | 162.64*** |
|  | (3.08) |
| Log relative price of F&V | -13.35*** |
|  | (3.41) |
| Household size | -18.11*** |
|  | (0.52) |
| Number of children under 5 | 17.56*** |
|  | (0.96) |
| Household head years of education | -1.36*** |
|  | (0.32) |
| Female headed households | 63.29*** |
|  | (4.23) |
| Rural location | 24.43*** |
|  | (2.60) |
| Agricultural households | 8.27*** |
|  | (1.80) |
| Hindu | 3.30 |
|  | (2.85) |
| **Caste (baseline: ‘other’ caste)**  *Scheduled Tribes* | -6.76* |
|  | (3.65) |
| *Scheduled Castes* | -8.94*** |
|  | (3.02) |
| *Other Backward Classes* | -5.17** |
|  | (2.54) |
| Observations | 98,868 |
| R-squared | 0.36 |

Standard errors in parentheses *** p<0.01, ** p<0.05, * p<0.1. Covariate set includes state dummy variables.

Appendix Table S4. RIF Unconditional Quantile Regression Results of drivers of F&V consumption (g/adult equivalent/day)

|  | (1) | (2) | (3) | (4) | (5) |
| --- | --- | --- | --- | --- | --- |
|  |  |  |  |  |  |
| Quantiles of the F&V consumption distribution | | | | | |
|  | 10th | 25th | 50th | 75th | 90th |
|  |  |  |  |  |  |
| Log per capita monthly consumer expenditure | 66.89*** | 86.12*** | 130.14*** | 199.85*** | 298.39*** |
|  | (2.05) | (1.72) | (2.12) | (3.43) | (7.16) |
| Log relative price of F&V | 4.69 | 8.00*** | -0.49 | -18.21*** | -41.07*** |
|  | (3.41) | (2.89) | (3.23) | (4.71) | (8.05) |
| Household size | -4.35*** | -8.42*** | -15.53*** | -24.58*** | -34.98*** |
|  | (0.48) | (0.43) | (0.52) | (0.80) | (1.47) |
| Number of children under 5 | 8.04*** | 11.39*** | 16.88*** | 19.84*** | 27.74*** |
|  | (1.35) | (1.19) | (1.33) | (1.73) | (2.60) |
| Household years of education | -0.38 | 0.02 | -0.56* | -1.17** | -2.73*** |
|  | (0.27) | (0.25) | (0.31) | (0.47) | (0.85) |
| Female headed households | 4.85* | 11.63*** | 24.53*** | 54.07*** | 154.77*** |
|  | (2.73) | (2.42) | (3.12) | (5.11) | (11.17) |
| Rural location | 8.57*** | 12.91*** | 15.82*** | 27.87*** | 54.84*** |
|  | (1.51) | (1.51) | (2.03) | (3.34) | (6.21) |
| Agricultural households | 5.96*** | 7.17*** | 7.37*** | 9.10*** | 13.05** |
|  | (1.67) | (1.50) | (1.84) | (2.86) | (5.33) |
| Hindu | 1.00 | 2.40 | 4.23* | 6.36 | 5.58 |
|  | (2.24) | (2.12) | (2.54) | (3.92) | (7.17) |
| Scheduled Tribes | -17.91*** | -10.33*** | -6.39* | -4.54 | 1.55 |
|  | (4.05) | (3.45) | (3.86) | (5.38) | (9.43) |
| Scheduled Castes | -2.97 | -2.80 | -7.31** | -10.34** | -17.06** |
|  | (2.65) | (2.45) | (2.95) | (4.44) | (8.09) |
| Other Backward Classes | 1.94 | 3.25* | 0.67 | -3.69 | -11.26 |
|  | (2.06) | (1.95) | (2.47) | (3.88) | (7.24) |
|  |  |  |  |  |  |
| Observations | 98,868 | 98,868 | 98,868 | 98,868 | 98,868 |
| R-squared | 0.11 | 0.20 | 0.26 | 0.25 | 0.17 |

Source: NSS (2011-2012). Robust standard errors in parentheses *** p<0.01, ** p<0.05, * p<0.1. Controls included but not reported are state dummy variables.

Appendix Table S5. Multilevel regression estimates of influence of district-level road and market infrastructure on household F&V consumption (grams/adult equivalent/day)

|  | (1) | (2) |
| --- | --- | --- |
|  | OLS with state dummy variables | Multilevel regression |
|  |  |  |
| Road density (km of road per 1000 km sq. of land area) | 19.06*** | -14.87^*^ |
| \| \|  \| .*** \| -13.90^*^ \| \| --- \| --- \| --- \| \| \| --- \| --- \| --- \| --- \| | (5.39) | (7.28) |
| Market density (number of agricultural markets per 1000 km sq. of land area) | 1.95*** | 4.59^***^ |
|  | (0.78) | (1.00) |
|  |  |  |
| Observations | 13,402 | 13,402 |

Source: NSS (2011-2012). Standard errors in parentheses *** p<0.01, ** p<0.05, * p<0.1. Regressions also control for household expenditure, household size, household head education, household head gender, rural location, religion and caste dummy variables.
